# *Pneumocystis jirovecii* Pneumonia in an Immunocompetent Japanese Man: A Case Report and Literature Review

**DOI:** 10.1155/2019/3981681

**Published:** 2019-03-07

**Authors:** Hiromi Ide, Yoshikazu Yamaji, Kazunori Tobino, Masanobu Okahisa, Kojin Murakami, Yuki Goto, Takuto Sueyasu, Saori Nishizawa, Kohei Yoshimine, Miyuki Munechika, Masafumi Oya, Yuka Hiraki

**Affiliations:** ^1^Department of Respiratory Medicine, Iizuka Hospital, Japan; ^2^Department of Respiratory Medicine and Infectious Disease, Graduate School of Medicine, Yamaguchi University, Japan; ^3^Department of Pathology, Iizuka Hospital, Japan

## Abstract

We herein report the case of a 37-year-old immunocompetent man who died from* Pneumocystis jirovecii* pneumonia (PCP). He was initially treated for an acute exacerbation of interstitial pneumonia; however, the elevation of the patient's serum (1-3) *β*-D glucan (BG) level suggested the possibility of PCP and sulfamethoxazole trimethoprim was added. A postmortem pathological examination and retrospective Grocott's methenamine silver (GMS) staining of the bronchoalveolar lavage fluid (BALF), which was obtained on the day of admission, revealed PCP. The present case suggests that it is essential to perform a BG assay and GMS staining of BALF specimens when patients show diffuse ground-glass opacity on chest computed tomography, regardless of their immune status.

## 1. Introduction

*Pneumocystis jirovecii* pneumonia (PCP) is a common opportunistic infection in patients with acquired immune deficiency syndrome (AIDS) [1]. In patients with human immunodeficiency virus (HIV), a low CD4+ lymphocyte count (<200 cells/*μ*L) is a risk factor for the development of PCP; underlying malignancies, organ transplantation and immunosuppressive medications are other risk factors. PCP in patients without any of these risk factors is extremely rare [2]. We herein report a case of PCP in an immunocompetent patient.

## 2. Case Presentation

A 37-year-old Japanese man presented to our hospital with a nonproductive cough of two weeks in duration. He did not have fever or dyspnea. He had a history of right hemiparesis, intellectual disability with pica, and symptomatic epilepsy caused by intracerebral hemorrhage, which occurred at two years of age. He had never smoked or consumed alcoholic beverages.

**Figure 1:**
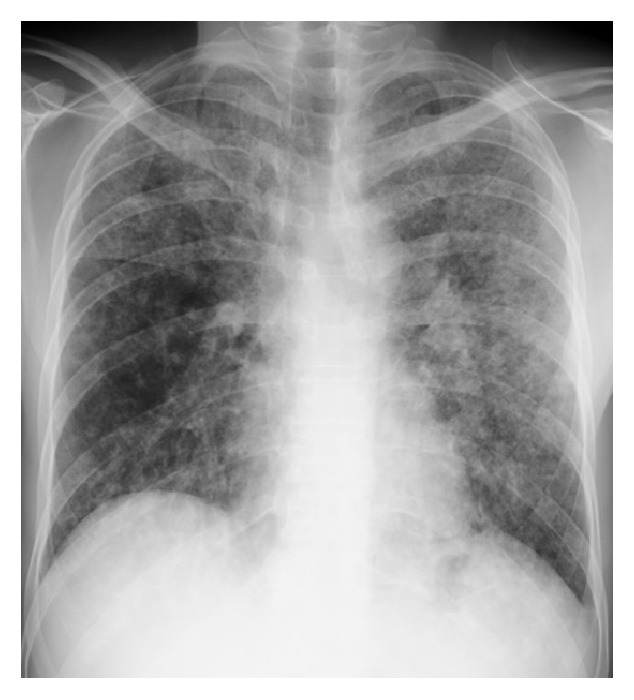
A chest radiograph obtained on the day of admission showed bilateral diffuse alveolo-interstitial infiltration, particularly in the left lung.

**Figure 2:**
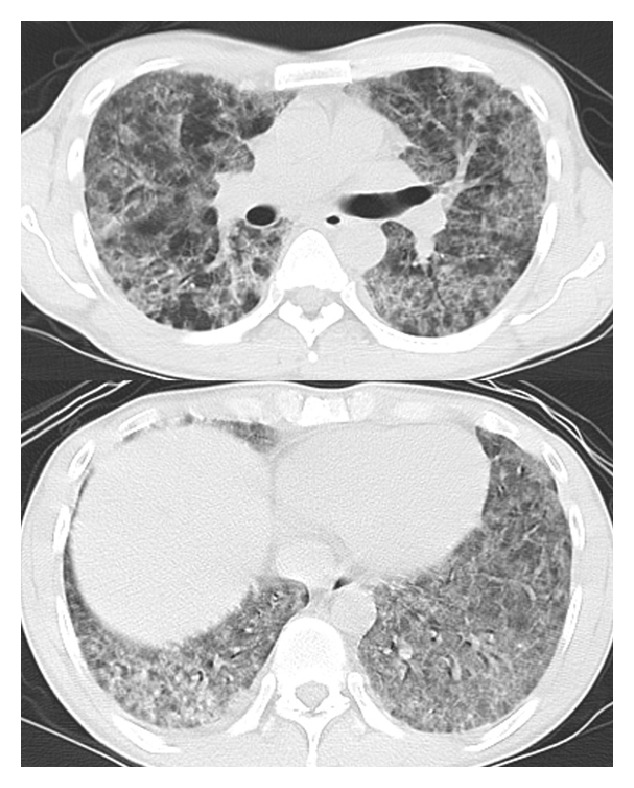
Chest computed tomography revealed bilateral airspace consolidation and ground-glass opacity.

His initial vital signs were as follows: blood pressure, 105/55 mm Hg; pulse rate, 70 beats/minute; respiratory rate, 18 breaths/min; SpO_2_, 93% in room air; body temperature, 36.5°C. There was no lymphadenopathy. Auscultation revealed no chest rales. A cardiovascular examination was normal, and no murmurs, rubs, or gallops were detected. Abdominal and neurological examinations were unremarkable, and the patient had no rash or petechiae. A chest radiograph revealed bilateral diffuse infiltration (Figure 1). Chest computed tomography (CT) revealed bilateral airspace consolidation and ground-glass opacity (Figure 2).

The patient's laboratory test values were as follows: hemoglobin, 14.9 g/dl; white blood cell count, 8,850/mm^3^ with a left shift; platelets, 329,000/mm^3^; serum aspartate aminotransferase 29 U/L (normal, 0-35 U/l); serum alanine aminotransferase, 31 U/L (normal, 0-35 U/l); serum lactate dehydrogenase, 425 U/L (normal, 119-229 U/l); serum total protein, 5.3 g/dl (normal, 6.5-8 g/dl); serum albumin, 2.1 g/dl (normal, 4-5 g/dl); serum C-reactive protein, 9.18 mg/dl (normal, < 0.2 mg/dl); serum KL-6, 2940 U/ml (normal, < 500 U/ml); serum surfactant protein D, 173.0 ng/ml (normal, < 109.9 ng/mL); and serum surfactant protein A, 115.0 ng/ml (normal, < 43.8 ng/mL). The patient's serum was negative for rheumatoid factor and antinuclear antibodies. The serum levels of immunoglobulin M, G, and A were within the normal ranges. An electrocardiogram revealed normal findings. An examination of the patient's sputum showed no predominant pathogen and no acid-fast organisms were observed on staining. Two sets of blood cultures were prepared at the time of admission; however, they did not yield any organisms.

The patient underwent a fiberoptic bronchoscopic examination that revealed a normal endobronchial system, and combined bronchoalveolar lavage (BAL)/transbronchial biopsy (TBLB) was performed. BAL fluid (BALF) was obtained from the right middle lobe. The results of the BALF analysis were as follows: histiocytes, 87%; neutrophils, 6%; lymphocytes, 4%; and eosinophils, 3%. Routine cultures of bronchial the washings were negative. The TBLB sample from the right upper lobe revealed alveolar septal thickening due to chronic inflammation, as well as collagen-type fibrosis.

After these examinations, the patient was diagnosed with acute interstitial pneumonia, and intravenous levofloxacin (500 mg) was administered once daily with corticosteroid pulse therapy (methylprednisolone [1000 mg] for three days) followed by prednisolone (1 mg/kg/day). On the 5th day after the initiation of therapy, his respiratory condition worsened and noninvasive positive pressure ventilation was started. His interstitial pneumonia was thought to be getting worse, and cyclophosphamide (500 mg/body) was administered intravenously. On the same day, an additional examination revealed the elevation of the serum (1-3) *β*-D glucan (BG) level (104.3 pg/ml; normal, <6.0 pg/ml). He was therefore suspected to have fungal infection or PCP, and voriconazole (200 mg, every 12 hours) and sulfamethoxazole trimethoprim (1600 mg and 320 mg, respectively, every 8 hours) were started. An additional HIV antibody test was negative and the serum protein electrophoresis revealed normal findings. Despite the additional antifungal therapy, his respiratory status gradually worsened, and intravenous corticosteroid pulse therapy (methylprednisolone [1000 mg] for three days) and cyclophosphamide therapy (500 mg/body) were each administered a second time, on the 8th and 11th days, respectively, without improvement. The patient died due to respiratory failure on the 12th day.

**Figure 3:**
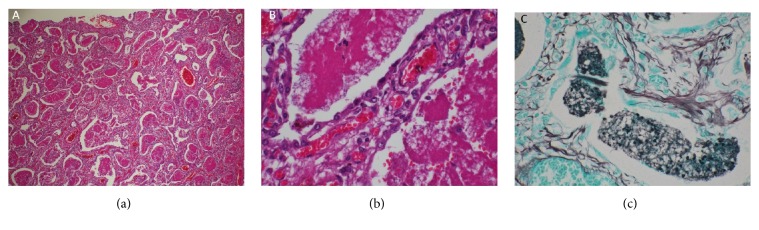
(a) A photomicrograph (original magnification, ×40; hematoxylin-eosin staining) of a section of lung tissue. The section showed homogeneous septal thickening and intra-alveolar eosinophilic exudate. (b) A high-power photomicrograph (original magnification, ×400; hematoxylin-eosin staining) of a section of lung tissue showed thickening of the alveolar septa due to both chronic inflammation and a small amount of collagen. The intra-alveolar spaces were filled with foamy acellular eosinophilic exudate. (c) A high-power photomicrograph (original magnification, ×400; Grocott's methenamine silver staining) of a section of lung tissue. Numerous cysts of* Pneumocystis jirovecii* were observed within the intra-alveolar foamy exudate.

**Figure 4:**
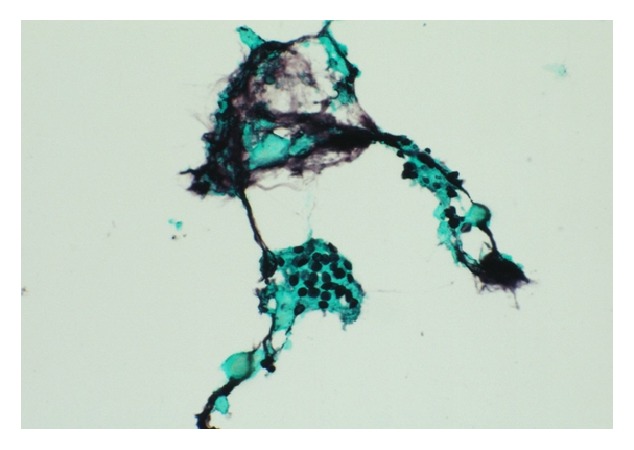
Grocott's methenamine silver staining of bronchoalveolar lavage fluid samples obtained on the day of admission revealed a small number of cystic forms of* Pneumocystis jirovecii.*

A postmortem pathological examination of the lung tissue demonstrated that the alveolar spaces were filled with foamy amorphous material composed of abundant numbers of cystic forms of* Pneumocystis jirovecii* and cellular debris, as well as an inflammatory reaction with hyaline membranes (Figure 3). Retrospectively, Grocott's methenamine silver (GMS) staining of the BALF and TBLB samples obtained on the day of admission revealed a small amount of the cystic form of* Pneumocystis jirovecii *(Figure 4). It was therefore thought that PCP had been present on the day of his admission.

## 3. Discussion

*Pneumocystis jirovecii *was first described by Chagas in 1909, but it was not shown to cause human disease until 1951. In Europe, an epidemic of PCP occurred in Europe in premature and malnourished children the late 1930s and 1940s. In the 1960s and 1970s, cases of PCP were seen in patients with congenital immune defects, acquired defects secondary to malignancy or its treatment, organ transplantation, and disorders that were treated with corticosteroids or cytotoxic drugs. However, PCP remained a relatively rare condition until 1981, when AIDS was described. Since then there has been a significant increase in the incidence of PCP, which has become the most infectious complication in patients with AIDS.

The risk of PCP is increased 5-fold in in HIV patients with a CD4+ lymphocyte count of ≤200 cells/*μ*L [7]. The other major risk factors for PCP in patients with and without HIV include—but are not limited to—a history of hematological malignancy, organ transplantation, inflammatory conditions, solid tumors, and the use of immunosuppressive drugs. Moreover the most significant risk factors for PCP in patients without HIV are glucocorticoid use and cell­mediated immunity defects, which lead to lung surfactant alterations, thereby predisposing the patient to pneumonia [8].

To the best of our knowledge, there are a few reports of PCP in patients without underlying immunosuppressive diseases (Table 1). In 1993, Cano et al. described five patients who developed PCP without any underlying conditions associated with immunosuppression; in their literature review, they only identified 11 other reported cases [3]. Since then, another three cases of PCP in immunocompetent patients have been reported [4–6]. In Cano's case series, two patients had previous or simultaneous infection by another microorganism (*e.g.*, a viral upper respiratory tract infection or* Mycoplasma pneumoniae*). It was hypothesized that of PCP might develop in patients in whose immunocompetence shows a transitory alteration due to a previous or concomitant infection. In fact, Mycoplasma is reported to lead to the polyclonal activation of B cells and the suppression of T cells, which would explain the appearance of transitory cutaneous anergy [9]. However, the precise mechanisms that lead to the development of PCP remain unclear.

A definitive diagnosis of PCP is made by the visualization of pneumocystis organisms on histopathological staining. The cell wall of the cysts can be visualized with GMS staining. The induction of sputum with hypertonic saline has a diagnostic yield of approximately 60% and should be performed as the initial diagnostic procedure, particularly in patients with AIDS [10]. Sputum induction may be less sensitive in patients without HIV infection because the immunodeficiency caused by HIV infection typically leads to a greater alveolar load of Pneumocystis organisms. Early fiberoptic bronchoscopy is recommended for patients in whom induced sputum is negative or from whom an induced sputum sample cannot be obtained. The diagnostic yield of BAL is >90%, and this may be increased if multiple lobes are sampled [10]. Open-lung biopsy is the most invasive procedure; however, shows very high sensitivity and specificity because it provides the greatest amount of tissue for the diagnosis [11].

We first suspected acute interstitial pneumonia based on the patient's symptoms, the physical examination findings, and the TBLB and CT findings. At first, the patient's BALF was not subjected to GMS staining (which subsequently revealed* Pneumocystis jirovecii *infection) because we considered that it was highly unlikely that* Pneumocystis jirovecii* was the causative organism based on the patient's immune status and past medical history. The high level of BG helped us to make a definitive diagnosis. BG is a cell wall component of all fungi. The serum is typically tested to determine the level of BG for the diagnosis of invasive* Aspergillus*; however, BG is also present in the cell wall in patients with PCP.

PCP is typically associated with high levels of BG. The levels should decline with effective therapy. The potential utility of this assay was illustrated in a retrospective case-control study of 295 patients with suspected PCP. Among these subjects, BALF specimens were observed by microscopy to detect PCP and serum samples were tested for BG [12]. In that study, the BG assay had a sensitivity of 92% and a specificity of 86% for detecting PCP. In our case, the serum BG level was 104.3 pg/ml (normal, <6.0 pg/ml); thus, we could consider PCP in the differential diagnosis and began to treat the patient accordingly. After treatment with sulfamethoxazole/trimethoprim, the value was decreased to 33.3 pg/ml, nevertheless we were unable to save the patient. A serum BG assay is helpful in the differential diagnosis for PCP, and should be performed whenever high-resolution chest CT reveals diffuse bilateral ground-glass attenuation, including reticulation, septal thickening and micronodules and air space consolidation in a patient with suspected IP, because healthy individuals can develop PCP, similarly to our case.

In conclusion, we experienced a case of PCP in a patient without any underlying risk factors. It may therefore be necessary to perform a serum BG assay and GMS staining of BALF specimens in patients with diffuse ground-glass opacity, regardless of the patient's immune status.

## Figures and Tables

**Table 1:** Review of previously published cases.

Author	Patient demographics	Initial presentation	Initial radiological findings	Means of diagnosis	Outcome
Cano [3]	39-year-old man	Fever and dyspnea	Left pleuraleffusion and bilateral interstitial infiltrate	GMS staining of TNA specimens	Survive
	30-year-old man	Fever and dyspnea	Diffuse alveolar infiltrate	GMS staining of BALF specimens	Survive
	37-year-old woman	Fever, cough, and malaise	Bilateral alveolointerstitial infiltrate	GMS staining of BALF specimens	Survive
	37-year-old man	Cough and purulent sputum	Bilateral nonhomogeneous alveolar infiltrate	GMS staining of TNA specimens	Survive
	55-year-old man	Fever and chill	Bilateral alveolar infiltrate in the middle and lower fields	GMS staining of BALF specimens	Survive
Koshy [4]	56-year-old man	Cough, dyspnea, and hemoptysis	Bilateral nonhomogenous opacities in the middle and lower zones	GMS staining of induced sputum	Survive
Harris [5]	51-year-old man	None	A right upper lung nodule	Open lung biopsy	Survive
Nejmi [6]	30-year-old woman	Productive cough and dyspnea.	Reticulo-micro-nodular opacities in the right lung	Induced sputum	Survive
Present case	37-year-old man	Non-productive cough	Bilateral diffuse infiltration	GMS staining of BALF specimens	Dead

PCP indicates *Pneumocystis jirovecii* pneumonia; BALF, bronchoalveolar lavage fluid; GMS, Grocott's methenamine silver; TNA, transthoracic needle aspiration; COPD, chronic obstructive pulmonary disease

## Data Availability

Data have been collected retrospectively from the patient record after he deceased.

## Abbreviations

BALF: Bronchioloalveolar lavage fluid
CT: Computed tomography
GMS: Grocott's methenamine silver
PCP: Pneumocystic pneumonia
TBLB: Transbronchial lung biopsy.
